# The Effect of Multi-Step Tempering and Partition Heat Treatment on 25Cr2Ni3MoV Steel’s Cryogenic Strength Properties

**DOI:** 10.3390/ma17020518

**Published:** 2024-01-21

**Authors:** Ye Chen, Ran Chen, Yanchen Yao, Na Min, Wei Li, Anna Diao

**Affiliations:** 1 Shanghai Key Laboratory of Material Laser Processing and Modification, Shanghai Jiao Tong University, Shanghai 200240, China; 2 Institute of Advanced Steels and Materials, School of Materials Science and Engineering, Shanghai Jiao Tong University, Shanghai 200240, China; 3 The State Key Laboratory of Metal Matrix Composites, Shanghai Jiao Tong University, Shanghai 200240, China; 4 Shanghai Marine Diesel Engine Research Institute, Shanghai 201108, China; 5 Key Laboratory for Microstructures, Shanghai University, Shanghai 200444, China

**Keywords:** 25Cr2Ni3MoV steel, multi-step tempering and partition, cryogenic mechanical property, CPFEM

## Abstract

In this study, the refinement of two microstructures was controlled in medium carbon 25Cr2Ni3MoV steel via multi-step tempering and partition (MTP) to achieve high cryogenic strength–ductility combinations. Microstructure evolution, the distribution of stress concentration, and microcrack formation and propagation during cryogenic Charpy impact testing were investigated. Compared with their performance in the quenching and tempering states (QT), the MTP steels showed a significant improvement in yield strength (1300 MPa), total elongation (25%), and impact toughness (>25 J) at liquid nitrogen temperature (LNT). The strengthening contributions mainly originated from the high dislocation density and refinement cementite (size: 70 nm) in the martensite lath (width: 1.5 μm) introduced by refined reversed austenite and its latter decomposition. The instrumented Charpy impact results indicated that cracks nucleated in the primary austenite grain (PAG) boundary for two steels due to the strain concentration band preferring to appear near PAGs, while cracks in the QT and MTP samples propagated along the PAGs and high-angle grain boundary (HAGB), respectively. The crystallized plasticity finite element simulation revealed that the PAG boundary with cementite precipitates of large size (>200 nm) was less able to dissipate crack propagation energy than the HAGBs by continuously forming a high strain concentration area, thus leading to the low-impact toughness of the QT steel.

## 1. Introduction

With the development of exploitation technology and the increasing demand for clean fuel, high-performance compressors and engines are emerging to adapt to lower-temperature environments, such as applications in liquefied natural gas fields [1,2,3,4]. It is necessary for the high-speed shaft steel used in such equipment to possess stricter cryogenic mechanical properties. Medium carbon Cr-Ni-Mo-V alloy steel, due to its high toughness, abrasive resistance, and hardness, is widely used to manufacture high-speed shafts [5,6,7]. However, its toughness drops as the temperature decreases [8], and the ductile–brittle transition observed is in line with the temperature-dependent moving ability of screw dislocations in the BCC structure of metals [9].

The method used to improve the toughness of single-phase BCC steel during tempering comprises two aspects: reducing the effective martensite grain size [10] and controlling the shape and size of cementite (decreasing the local stress concentration) [11]. During tempering, martensite microstructures mainly manifest as the low-angle grain boundary (LAGB), vanishing in ferrite, and the high-angle grain boundary (HAGB), remaining due to the pinning of precipitates [12]. Reducing the tempering temperature and shortening the tempering time can effectively help to maintain the grain size and strength of quenching martensite. However, controlling the growth of carbide particles requires an element diffusion balance between the matrix and carbide, and the interface can increase the diffusion rate and accelerate abnormal growth. Although elements with large atomic numbers in cementite (M_3_C, M = Mo and V) can effectively block the interface migration rate from the cementite to the matrix by the solute drag effect [13], the coarsening phenomenon still occurs at high-temperature tempering [14], especially cementite spheroidization.

The concentrated distribution of carbide neighboring the grain boundary also has a negative effect on toughness because it promotes potential microvoid nucleation [15] and stress concentration, according to the simulation results of damask software [16]. The inhomogeneous strain distribution at interfaces is the origin of ductility reduction [17]. Therefore, the competition between the controlled recovery of martensite to maintain its strength and relatively uniform small-sized and round-shaped carbides during tempering restrict the comprehensive strength/toughness properties of these types of steel. Sachin Kumar [18] utilized cementite containing austenite-stabilizing elements as a nucleation point for martensite reversion transformation, finally forming reversed austenite containing bulk cementite after cooling at room temperature. The cryogenic stability of reversed austenite will decrease when the corresponding total volume fraction or the volume of the single austenite grain exceeds the critical value due to dilution of the austenite stable element [19]. Recently, G.G. Ribamar [20] induced blocky austenite to decompose while tempering at 500 °C to obtain small dispersed carbides and film ferrite. On the other hand, E. Tkachev [21] improved the cryogenic impact toughness by promoting cementite spheroidization (η-Fe_2_C (rod-like)→Fe_3_C (round-like)) after tempering at 500 °C.

Dislocation density, grain size, and nanoprecipitation characteristics all contribute to the strength and toughness of materials. The present study aimed to elucidate the effect of multi-tempering and partition (MTP) on both the refinement of martensite lath and the dispersion of carbide, as well as the corresponding influence on the cryogenic properties of 25Cr2Ni3MoV steel. Moreover, the MTP process was designed to activate the martensite reversion transformation by nucleating carbides. The decomposition of thin-film reverted austenite was hypothesized to refine the cementite size along the PAG boundary. Finally, the strengthening contribution and crack propagation behavior were addressed based on the multiple mechanisms and strain distribution correlated with the result of the crystallized plasticity finite element model (CPFEM).

## 2. Experimental Procedures

**Figure 1:**
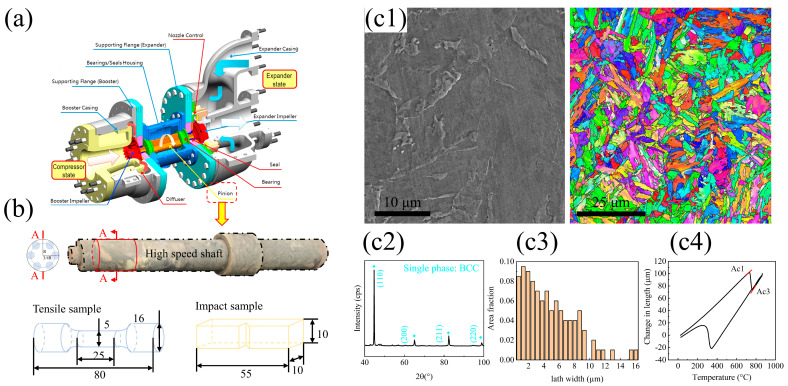
Materials and experimental details for the 25Cr2Ni3MoV steel. (**a**) The gas compressor schematic diagram for liquefied natural gas; (**b**) the high-speed shaft was fabricated through the hot forging of the corresponding sample preparation of the tensile and impact tests (units in mm); and (**c**) the primary microstructure of the experimental shaft after hot forging, including morphology (**c1**), phase composition (**c2**), grain size distribution (**c3**), and phase transformation point (**c4**) (Ac1: the phase transformation temperature from ferrite to austenite; Ac3: the full austenite region).

The material was provided by the China State Shipbuilding Corporation (CSSC, Shanghai, China), and this material is widely used in shafts (Figure 1a). It is manufactured by combining annealing and multi-pass forging as an initial state. The initial-state microstructure is single-phase martensite (Figure 1(c2)), and the lath size distribution follows a Gaussian distribution with a mean value of around 2.1 μm (Figure 1(c3)). The shaft, in this case, is medium carbon 25Cr2Ni3MoV steel, and the corresponding chemical composition is shown in Table 1.

It is well-known that the starting temperature (Ac1) and completion temperature (Ac3) for austenitization can be extracted from the tuning spot in the thermal expansion curve (Figure 1(c4)). According to the results above, two heat treatment processes were designed for the shaft. (i) QT: The shaft was austenitized at 900 °C for 4 h, quenched into water, and then tempered for 10 h at 600 °C. (ii) MTP: Firstly, the shaft was re-austenitized at 900 °C for 1 h and quenched into water; secondly, the shaft was multi-tempered and partitioned by S1, S2, and S3 from 500 °C to 700 °C for 5 h in total.

Figure 1b shows the positions (where R is the radius of the shaft) from which the tensile and impact test samples were extracted and the corresponding three dimensions of the tested samples. Dog bone-shaped specimens with gauge dimensions of diameters of 5 mm, lengths of 25 mm, and V-notch samples of 55 mm × 10 mm × 10 mm were cut from the prepared steels along the axial direction using an electron discharge machine (APL250, Yokohama, Japan).

Uniaxial tensile with a strain rate of 0.5 mm/min (using an INSTRON 5565 tensile testing machine) was performed to measure the mechanical properties at room and liquid nitrogen temperature (−196 °C). The Charpy impact test (Zwick/Roell, 450 J impact tester, Ulm, Germany) was implemented at temperatures ranging from −196 °C to −140 °C. Rockwell hardness measurements were acquired by averaging ten measurements at room temperature. The microstructure was characterized using a JSM-7001F scanning electron microscope (SEM) after mechanical polishing and etching (4% Nital). Electron backscattering diffraction (EBSD) with an orientation imaging microscope system was employed on the SEM (Tescan, Mira, Brno, Czech Republic) to investigate the effective grain size and grain boundary characteristics after electropolishing using an electrolyte consisting of 10 vol.% perchloric acid and 90 vol.% alcohol. Aztech 2.1 software was used to process the EBSD data and images. The TEM samples were prepared by a twin jet electro-polisher at 26 V and −20 °C in an 8% perchloric and 92% alcohol solution and observed by a transmission electron microscope (JEOL, JEM 2100F, Tokyo, Japan) at 200 kV. X-ray diffraction analysis (Zeiss Crossbeam 550, Jena, Germany) carried out using an X-ray diffractometer with Cu-Kα radiation determined the phase components of the specimens. Next, 2θ angles from 40° to 100° were scanned at 40 kV with a step of 0.02°. The phase transformation behavior was certified by thermal dilatometry (DIL805A, TA Instruments, New Castle, DE, USA) using the tangent method, and corresponding phase content variation was analyzed using the saturation magnetization (SM) method through the use of a quantum-designed physical property measurement system (PPMS-9T (EC-II)).

## 3. Simulation Procedures

A crystal plasticity framework was used to simulate the deformation of the martensite lath grains, corresponding to displacement-controlled uniaxial tension at a quasi-static strain rate of 10^−3^ s^−1^ and room temperature (*T* = 23 °C) imposed on the generated representative volume elements (RVEs).

### 3.1. Deformation Kinematics

Mechanical response during uniaxial tension was simulated through the use of the finite element software Abaqus 2017, with a user material (UMAT) subroutine programmed based on the *continuum* crystal plasticity theory. The crystal lattice structures of martensite and cementite were body-centered cubic (BCC) structures and perfectly elastic states, respectively. The total deformation gradient tensor *F* can be decomposed into a component *F_e_*, representing elastic stretching and rigid body rotation, and a plastic component *F_p_* ($F=F_{e}\cdot F_{p}$). The plastic velocity gradient *L_p_* can be defined as the multiplication between the time derivative ${\dot{F}}_{P}$ and inverse matrix $F_{P}^{-1}$ (${L_{p}=\dot{F}}_{P}\cdot F_{P}^{-1}$).

### 3.2. Slip Deformation

The plastic velocity gradient $L_{p}$ is related to the slipping rate ${\dot{\gamma }}_{\alpha }$ of the corresponding slip system by:

(1) $${\dot{F}}_{P}\cdot F_{P}^{-1}=\sum_{\alpha }{\dot{\gamma }}_{\alpha }\cdot S_{0,\alpha }⊗{\;m}_{0,\alpha }$$

where the sum ranges over all activated slip systems and unit vectors $s_{0,a}$ and $m_{0,a}$ are the slip direction and normal to slip plane in the reference configuration. Plastic deformation comprises the motion of dislocations along slip planes; this study mainly considers the motion of 24 slip systems comprising both the primary {110}<111> slip system and the secondary {211}<111> slip system for martensite in the current sample.

The plastic slip rate ${\dot{\gamma }}_{\alpha }$ follows the classic flow rule [22]:

(2) $${\dot{\gamma }}_{\alpha }={\dot{\gamma }}_{0}\;sgn(\tau_{s}^{\alpha }){\left\{\left|\frac{\tau_{\alpha }}{g_{\alpha }}\right|\right\}}^{n}$$

where *n* is the rate-sensitivity exponent of slip, ${\dot{\gamma }}_{0}$ is the reference slip rate, and $\tau_{\alpha }$ is the resolved shear stress. $g_{\alpha }$ is the current slip resistance, whose initial value is usually the critical resolved shear stress (CRSS) of the *α* slip system, as follows:

(3) $${\dot{g}}_{\alpha }=\sum_{\beta }{h_{a\beta }\dot{\gamma }}_{\beta }$$

where ${\dot{\gamma }}_{\beta }$ is the plastic slip rate of the *β* slip system and $h_{\alpha \beta }$ is the slip hardening matrix, which satisfies the following equation:

(4) $$h_{a\beta }=qh_{aa}=-qh_{0}{sech}^{2}\left|\frac{h_{0}\gamma_{a}}{g_{\infty }-\tau_{0}}\right|$$

where $h_{0}$ is the initial hardening modulus, $\tau_{0}$ is the CRSS of the slip system and $g_{\infty }$ is its saturation value, and *q* is the ratio of the latent hardening modulus *h_αβ_* (*α ≠ β*) to the self-hardening modulus *h_αα_*. Parameter *q* is set to 1.4 [22] for a non-coplanar slip system because the BCC structure has several slip systems, including multiple slip surfaces.

### 3.3. Polycrystalline Geometric Based on EBSD

The grain shapes and crystallographic orientations of the RVE microstructure were generated from the EBSD measurements using MTEX 5.10 and Matlab 2022 software. A two-dimensional geometric model was meshed with a total of 506,944 elements. The mesh size was 0.07 μm, the same as the EBSD step size.

In this paper, the boundary conditions employed for uniaxial tensile simulation were consistent and are listed below. The node on the left was constrained, as there was no displacement in the X direction (u_x_ = 0), and the node on the right boundary was subjected to displacement, corresponding to the max. uniform strain from the experimental result (inset in Figure 10(b1)).

Based on preliminary research, the martensite in low-carbon steel produced after high-temperature tempering treatment is similar to ferrite, leading to the parameter of tempering martensite being closer to ferrite [23]. Thus, this study adopts the parameters of a ferrite matrix as the foundation [17]. Due to the higher dislocation density and the refined cementite in the MTP sample, the kinetics of dislocation slip were slightly modulated [24]. The crystal material parameters are primarily included, as shown in Table 2.

## 4. Results

### 4.1. Microstructure Characterization

**Figure 2:**
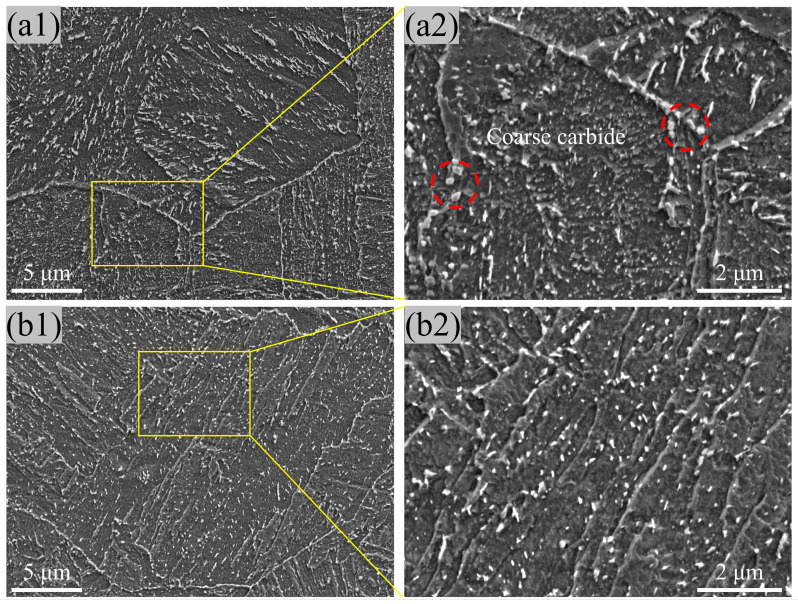
SEM image of 25Cr2Ni3MoV steel with the QT (**a1**,**a2**) and MTP (**b1**,**b2**) treatment.

Figure 2 shows the morphologies of the two types of steel observed using the SEM. The main microstructural constituents are tempering martensite and carbide, where the dark matrix is tempering martensite and the white particles are carbide. The coarse carbide particles were mainly located at the prior austenite grain (PAG) boundary (Figure 2(a2)) by the spheroidization growth; inversely, the transgranular carbide was the ellipse carbide with a high aspect ratio (6–8). A similar carbide distribution was obtained in the warm-rolling high-carbon steel [12]. On the contrary, the interface of martensite laths in the MTP sample was sharper; meanwhile, no granular carbide was obtained in the PAG boundary, and transgranular carbide was dispersed along the inter-lath with a low aspect ratio (1–3) (Figure 2(b2)).

**Figure 3:**
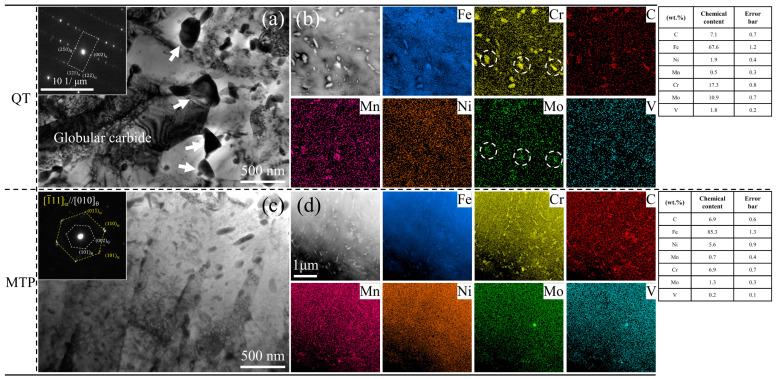
Precipitation of cementites in the martensite matrix following QT and MTP treatment. (**a**) BF image of the coarse θ-cementite in the QT samples; their average size is approximately 300 nm. (**b**) The element distribution mapping of coarse θ-cementite in the QT samples, with corresponding local EDS results gained through the use of point analysis. (**c**) BF image of the refined θ-cementite in the MTP samples; their average width is approximately 150 nm. (**d**) The element distribution mapping of the refined θ-cementite in the MTP samples, with corresponding local EDS results gained through the use of point analysis.

Both carbides in the two samples were certified as θ-cementite (M_3_C where M: randomly dispersed Fe, Cr, Ni, and Mo) through selected area electronic diffraction (SAED) and scanning transmission electron microscopy/energy-dispersive spectrometry (STEM/EDS), as shown in Figure 3. The average size of the coarse θ-cementite particles along the PAG boundary is more than 300 nm in the QT specimen, as indicated by the white arrow in Figure 3a. The transgranular θ-cementite in the MTP specimen is only half the size of the carbide in the QT, with a grain size of 150 nm, as shown in Figure 3c. The fine θ-cementite in the MTP sample has higher Ni content and lower Cr and Mo content (Figure 3b,d). The coarse cementite often accompanied the enrichment of Cr and Mo due to the enhancement of mobility in cementite when partitioned at 600 °C, according to the local equilibrium (LE) deduced by Y.X. Wu [14].

**Figure 4:**
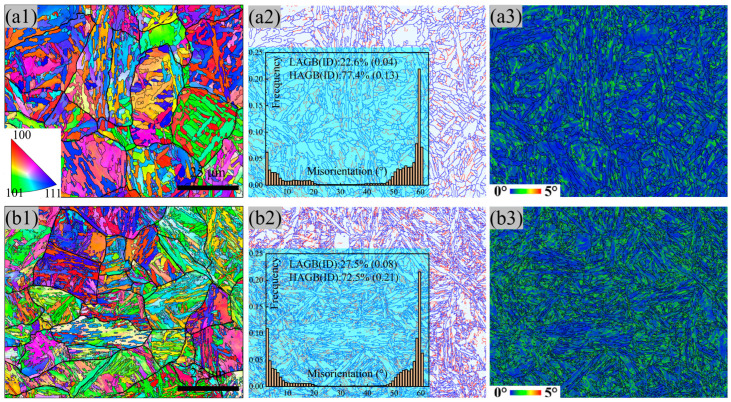
Inverse pole figure (IPF) image with the reconstructed prior austenite grain (PAG) boundary, the KAM image, and the grain boundary of 25Cr2Ni3MoV steel with the QT (**a1**–**a3**) and MTP (**b1**–**b3**) samples (LAGB: low-angle grain boundary (2°–15°), HAGB: high-angle grain boundary (>15°), and ID: interface density (μm^−1^)).

The microstructure feature of the two types of steel was determined using the EBSD method (Figure 4). The average sizes of prior austenite grains (PAGs) and the width of martensite lath (12 μm and 1 μm) in the MTP sample are smaller than the QT sample (20 μm and 4 μm), according to the results shown in the inverse pole figure (IPF) map (Figure 4(a1,b1)). The total interface density (ID is the ratio between the whole length of the interface and total area), attributed to martensite transformation, is 0.17 μm^−1^ and 0.29 μm^−1^ for the QT and MTP samples, respectively, and the corresponding proportion of LAGB attributed to dislocation recovery is 22.6% and 27.5%, as shown in Figure 4(a2,b2). It should be noted that the value of kernel average misorientation (KAM) is higher neighboring the LAGB (Figure 4(a3,b3)), which is related to misorientation induced by greater dislocation densities and more pronounced strain concentrations [25]. Therefore, the martensite laths with a greater dislocation density were obtained in the MTP sample.

### 4.2. Mechanical Properties

**Figure 5:**
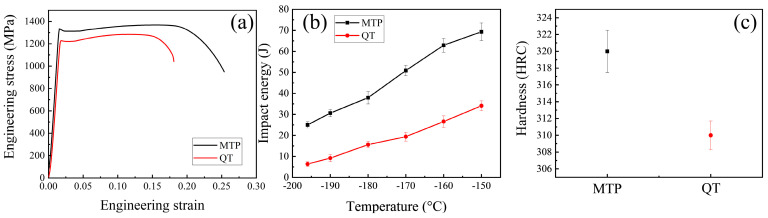
Mechanical properties of 25Cr2Ni3MoV steel: (**a**) tensile curves at −196 °C; (**b**) impact properties at different temperatures; and (**c**) hardness.

Figure 5a shows the engineering stress–strain curves tested at −196 °C. The comprehensive mechanics increased with the decreased grains and precipitated phase size due to grain refinement strengthening and the low-stress concentration. The MTP sample achieved a highest yield strength of 1336 MPa, an ultimate tensile strength of 1367 MPa, and an elongation of 29% (Table 3). Simultaneously, the impact toughness of MTP steel (25.4 J, −196 °C) is higher than the QT sample (7 J, −196 °C) at various temperatures (Figure 2b). The Rockwell hardness for the MTP and QT samples are depicted in Figure 5c, and they show a similar strength change trend. Accordingly, dual matrix and precipitated phase refinement markedly affect the low-temperature strength without sacrificing impact ductility.

### 4.3. Deformation Microstructure

**Figure 6:**
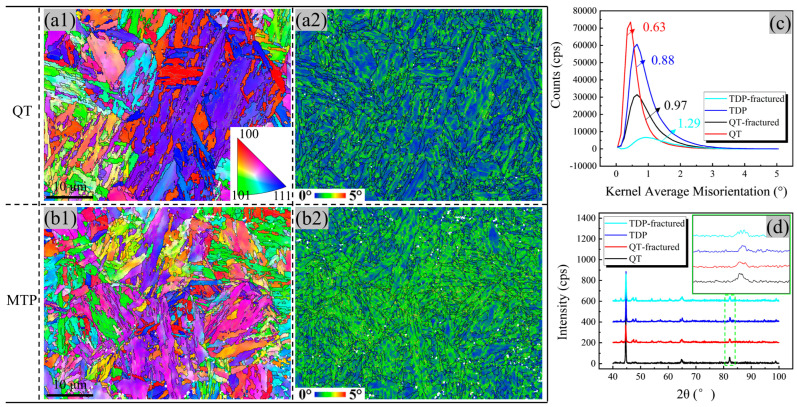
The microstructure of 25Cr2Ni3MoV steel after tensile fracture: (**a1**,**b1**) IPF image; (**a2**,**b2**) KAM distribution image; (**c**) KAM values of the QT and MTP samples in undeformed and fractured states; and (**d**) the XRD pattern of the QT and MTP samples in undeformed and fractured states; the inset in (**d**) shows the apparent diffraction peak broadening phenomenon owing to dislocation formation.

Figure 6 shows the EBSD image after the tensile fractured state of the two samples at −196 °C. The IPF map (Figure 6(a1,b1)) demonstrates that no deformation texture of martensite was formed in the two steels during tensile deformation at liquid nitrogen temperature (LNT). By comparing the KAM maps (Figure 6(a2,b2)), two results can be obtained: (i) a strain hardening behavior originating from dislocation pileup and multiplication was found in both samples and (ii) the average KAM value of the MTP sample is slightly higher than the QT sample (Figure 6c). To macroscopically estimate the dislocation increment state, the XRD method was applied to calculate the dislocation density using the Williamson–Hall (WH) formula [26], as follows:

(5) $$\beta_{i}=\frac{1}{D_{v}}+2\varepsilon S_{i}$$

(6) $$\rho =\frac{3\sqrt{2\pi }{\langle \dot{\varepsilon }\rangle }^{1/2}}{D_{v}b}$$

$\beta_{i}=\beta_{i}^{0}\cos\left(\theta_{i}\right)/\lambda$ represents the integral breadth of the diffraction peak in the reciprocal space, $\beta_{i}^{0}$ is the integral breadth of the diffraction peak,$\;\theta_{i}$ is the Bragg angle, λ is the wavelength (1.54056 Å), and $S_{i}=2\sin\left(\theta_{i}\right)/\lambda$ represents the length of the diffraction vector for the diffraction peak. 

The result in Figure 6d exhibits the XRD pattern of undeformed and fractured samples in the two samples, which includes corresponding diffraction peaks, referring to the (110), (200), and (211) crystallographic planes of the bcc phase. An obvious diffraction peak broadening phenomenon appears in both types of steel. According to the diffraction peak broadening value obtained in the inset, the values of dislocation density in the matrix were 2.38 × 10^14^ m^−2^ (QT) and 3.91 × 10^14^ m^−2^ (MTP). The values of dislocation density of deformation were 4.87 × 10^14^ m^−2^ (QT) and 5.92 × 10^14^ m^−2^ (MTP). The result of the XRD analysis shows almost the same trend as the value of KAM.

**Figure 7:**
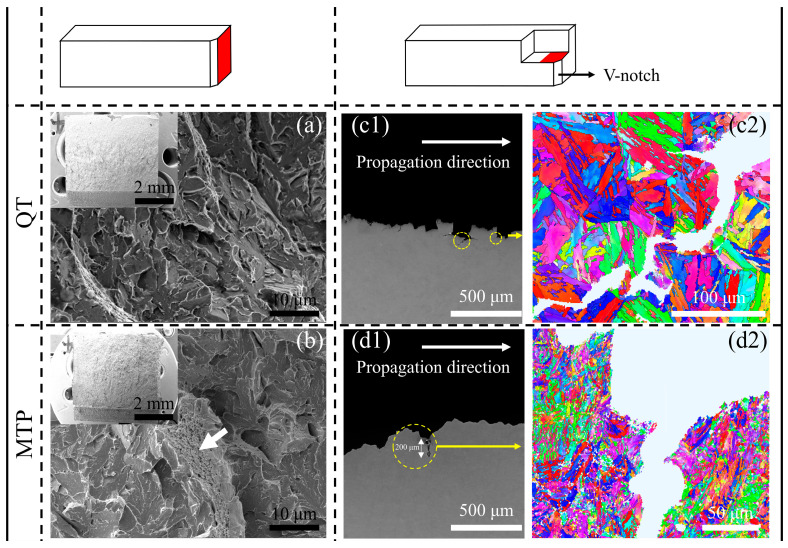
Fractography morphology of the QT and MTP samples after the impact test: (**a**,**b**) the fracture morphology; (**c1**,**d1**) the local profile fractography; and (**c2**,**d2**) the crack propagation trace determined by EBSD (the red area is the positions for the SEM in the Charpy samples).

Figure 7 demonstrates the fractured morphology and crack propagation path during the impact test at −196 °C for the two types of steel. A typical brittleness fracture with many cleavage river patterns was obtained in the two samples. Interestingly, the local impact fracture of the MTP samples has a mixed feature combining quasi-cleavage and dimples along the tear ridge, as shown by the white arrow (Figure 7b), but no dimples were discovered in the QT fracture (Figure 7a). Although the tortuous propagation paths of the main crack occurred in both types of steel, the secondary crack propagation in the QT sample extended along the PAG boundary with a length of less than 120 μm (Figure 7(c1,c2)) compared to the HAGB in the MTP sample with a length of more than 160 μm (Figure 7(d1,d2)). Thus, compared to MTP steel, QT steels have a recognizable intergranular fracture face along the PAG boundary.

## 5. Discussion

### 5.1. Microstructure Evolution during MTP Heat Treatment

**Figure 8:**
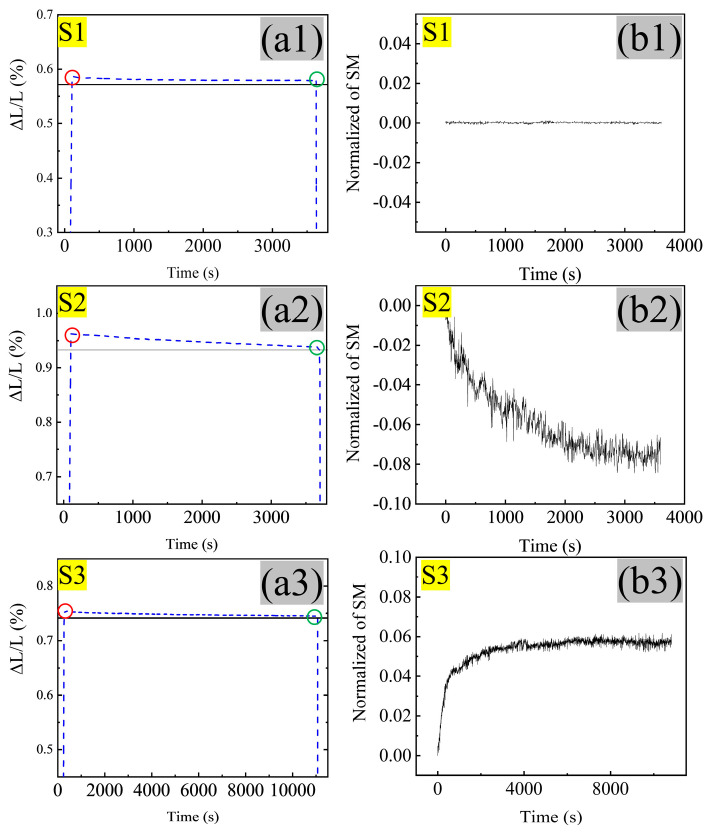
Phase transformation behavior of 25Cr2Ni3MoV steel during the isothermal stage in the MTP process: (**a1**) the DIL curve showing the length change for S1; (**a2**) the DIL curve showing the length change for S2; (**a3**) the DIL curve showing the length change for S3 (ΔL/L starts from 0%, and the starting and ending of the isothermal stage are indicated by the red and green circles); and (**b1,b2,b3**) the variation in SM during the isothermal stage for S1, S2, and S3.

The DIL was scheduled to capture the phase transformation behavior during the MTP process (Figure 8(a1,a2,a3)). No martensite transformation occurs at each step (S1, S2, and S3), as the temperature falls to room temperature when the cooling rate is 50 °C/s. Furthermore, the corresponding change in length (ΔL/L) at the isothermal stage of every step shows a decreasing trend with the extension of isothermal time. SM was measured, as shown in Figure 8(b1,b2,b3), to analyze phase transformation and the carbide precipitation phenomenon further.

**Figure 9:**
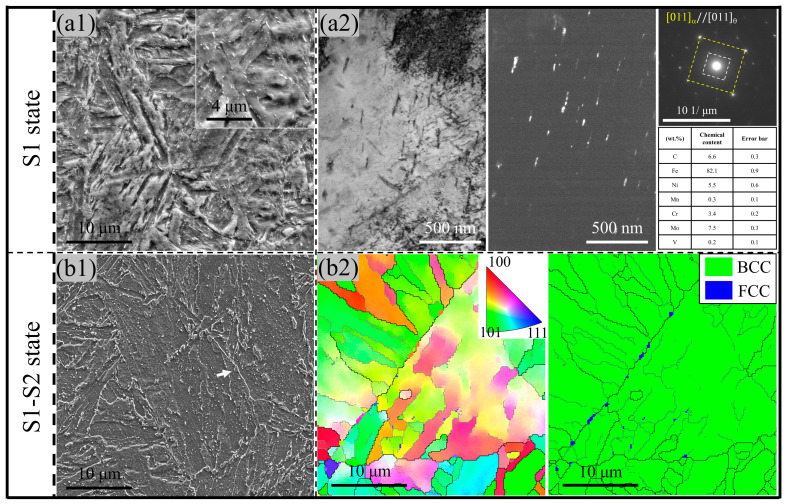
Microstructures of 25Cr2Ni3MoV steel after the S1 state (**a1,a2**) and the S2 state (**b1,b2**): (**a1**) SEM image showing the tempering martensite and nano carbide as the inset and (**a2**) the bright field (BF) and dark field (DF) image for globular cementite and the corresponding element content determined using the single-point energy-dispersive spectroscopy (EDS) method. (**b1**) SEM image showing the austenite. (**b2**) EBSD maps of austenite along the high-angle grain boundary.

Comparing Figure 1b and Figure 9(a1), the decrease in length in S1 (Figure 8(a1)) with a stable SM value (Figure 8(b1)) was attributed to the lamellar cementite precipitation (width: 10 nm; length: 70 nm), which was generated from the interaction between dislocation in the quenching martensite and carbide segment.

The decrease in length in S2 (Figure 8(a2)) with a decreasing SM value (Figure 8(b2)) is related to the occurrence of transformation between different crystalline structures [27], i.e., a small degree of reversion austenite formation, accompanied by Ni, Mn, and C element partition from martensite into austenite. This can be inferred from two perspectives: (i) the SM value decreasing due to austenite as a non-magnetic phase, whose presence is often accompanied by magnetic decline [28], and (ii) the retained austenite along the HAGB after cooling (Figure 9(b2), which is the result of hydrostatic pressure and high stability [29]. Notably, the martensite reversion transformation happened below Ac1. This is attributed to the precipitation of cementite with high Ni content (Figure 9(a1,a2)) during the S1 stage, which not only provided the revision austenite nucleation site but more carbon and nickel elements from the dissolution of cementite can also improve the local driving of reversion martensite transformation and promote the migration of the interface from austenite to martensite [30].

The decrease in length in S3 (Figure 8(a3)) with an increasing SM value (Figure 8(b3)) was connected to austenite decomposing during tempering [31], with the corresponding product including ferrite and fine lamellar cementite (Figure 2b) from the austenite decomposition inherited from the higher Ni content (Figure 3d) rather than spheroidized cementite with high Cr and Mo content. This can effectively reduce the coarsening of cementite at the HAGB. A similar phenomenon was observed in high carbon-bearing steel containing blocky austenite after tempering [20]. The dislocation as a lattice defect has a higher binding energy for the carbon element segment [32], leading to an intragranular uniform distribution of carbon elements rather than only segregation at the grain boundary. Meanwhile, the kinetics of cementite growth from the martensite during the tempering is directly proportional to the relationship with the local carbon concentration. Consequently, the cementite in the MTP sample was precipitated from martensite, which is more refined than the QT sample due to the higher dislocation density.

Compared to the QT sample, the MTP process promotes the refinement of martensite lath. The long tempering time and high tempering temperature led to grain growth due to the merging of the subgrain boundary, i.e., the vanishing of the LAGB due to dislocation recovery [18]; thus, the lower dislocation density was retained in the martensite lath of the QT sample (Figure 4(b3)).

### 5.2. Strengthening Contribution

The strengthening contribution in this experimental steel mainly involves intrinsic strengthening (*σ_INT_* = *σ*_0_ (lattice friction stress) + *σ_SS_* (solid solution strengthening)) [33], grain boundary strengthening (*σ_GB_*) [34], precipitation strengthening (*σ_PRE_*) [35], and dislocation strengthening (*σ_DIS_*) [26], and the corresponding calculation formula is as follows:

(7) $${\Delta \sigma }_{GB}=K\times (1/\sqrt{d})$$

(8) $$\Delta \sigma_{PRE}=0.538Gbf^{\frac{1}{2}}/\bar{d}\cdot ln\left(\bar{d}/2b\right)$$

(9) $$\Delta \sigma_{DIS}=M\alpha Gb\sqrt{\rho }$$

where *K* is the strengthening coefficient, *d* is the average width of martensite lath, *f* is the area fraction of cementite, $\bar{d}$ is the average size of cementite, *b* is the Burgers vector, *M* is the average Taylor factor, α is the interaction strength between dislocations, and *G* is the shear modulus. The *σ_INT_* is 160 MPa.

From the results in Table 4, it can be seen that the double refinement of the matrix and precipitated phase promoted strength improvement before yield strength. Meanwhile, dislocation multiplication and pileup provided a working hardening ability during cryogenic deformation in the MTP sample.

### 5.3. Deformation Compatibility of Tempered Martensite and Precipitate

The microstructural evolutions and associated mechanical behavior of the 25Cr2Ni3MoV steel during deformation can be depicted using the CPFEM.

**Figure 10:**
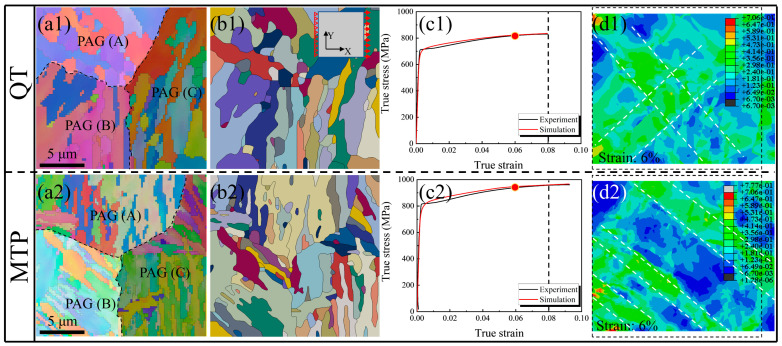
Experimental and simulated results for QT and TDP steel at room temperature: (**a1**,**a2**) IPF maps with three PAG boundaries. (**b1**,**b2**) The orientation map used for the CPFEM simulation. (**c1**,**c2**) True stress–strain curve, comparing the experimental and simulated observations. (**d1**,**d2**) The accumulated shear strain distribution at a true strain of 6%; note: in the cloud pictures, the strain ranges are set to 0.0067–0.706, locations with values exceeding the maxima are shown in gray, and those with values less than the minima are shown in black.

Figure 10(a1,a2,b1,b2) show the simulated IPF images of QT and MTP steel, corresponding to the EBSD measurements with the junction of the three PAG boundaries; the different colors indicate the variations in the crystal orientations among different martensite variants. Furthermore, the significant difference between QT and MTP steel in terms of grain size, crystal orientation, and morphology prohibited comprehension.

There is a slight difference between the experimental and simulated true stress–strain curve, as there is a yield plateau on the experimental tensile curve because of the existence of plastic, affecting the stability performance at the initial deformation. However, this does not affect the application of the model at present because the yield strength and subsequent hardening trends were in good agreement with the experimental curve (Figure 10(c1,c2)).

Figure 10(d1,d2) shows the accumulated plastic shear strain distribution of QT and MTP steel at 6% true strain. Although the accumulated plastic shear strain degree of the two samples is similar, the strain concentration band in the QT sample (the area for the white frame line) is wider than the MTP sample. Meanwhile, cross-distribution occurs at neighboring PAG boundaries in the QT samples, which can become a crack nucleation point and aggregate the local deformation concentration, resulting in incompatibility deformation [36]. Conversely, the existence of parallel and dispersive strain concentration bands in the MTP sample can prove the formation of geometry necessary dislocation (GND) caused by the high strain gradient, i.e., higher dislocation strength was achieved [37].

**Figure 11:**
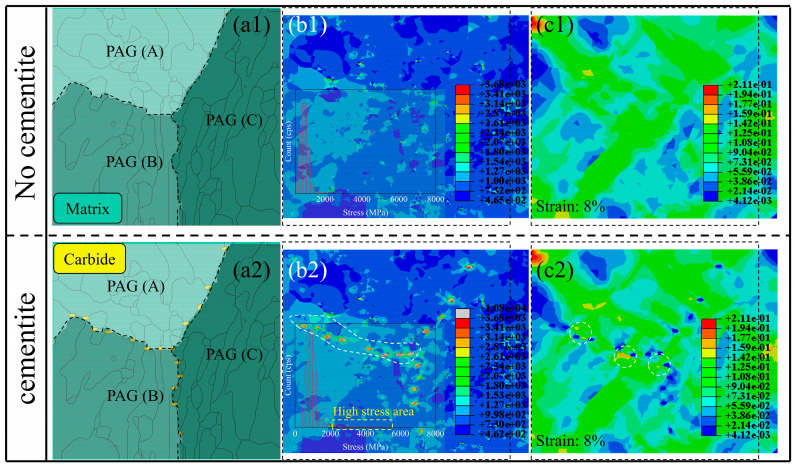
CPFEM results for QT steel considering the cementite along the PAG boundary: (**a1**,**a2**) phase maps with three PAG boundaries. Note: dark green regions represent the matrix (tempering martensite), and yellow regions represent cementite. (**b1**,**b2**) The stress distribution map with the ranges is set to 460–3680 MPa. Locations with values exceeding the maxima are shown in gray. (**c1**,**c2**) The strain distribution map.

The new model was introduced by adding coarse carbide particles near the PAG boundary based on the QT model (Figure 10(a2)), as shown in Figure 11(a2). Compared to the no-cementite model (Figure 11(a1)), the high-stress area mainly concentrates on the interface between the cementite and matrix (Figure 11(b2)). In comparison, the high-stress area was connected to a curve along the PAG boundary, especially along the tensile direction, as shown in the area of the white frame in Figure 11(b2); simultaneously, the formation of the local strain concentration area with the spacing of the precipitated phase decreasing can be seen in Figure 11(c2).

As is known, the microvoid can easily nucleate between the soft and hard phases due to deformation incompatibility. In this study, however, the microvoid occurred between the same phases (tempering martensite lath), as shown in Figure 7(c2) due to the occurrence of strain concentration. That is to say, damage nucleation during plastic deformation was aggravated by the strain concentration between two cementites rather than the stress concentration induced by dislocation pileup.

On the other hand, the ability of PAG boundaries to dissipate crack propagation energy was weakened due to the continuous high-stress area and promoted cracking between two PAGs, compared to other HAGBs. The deformation that occurs during the impact condition is more drastic than that which occurs during the uniaxial tensile condition; thus, the QT model under the tensile condition can explain the deterioration in fracture toughness to a certain extent. The crack shows a preference for propagating along the PAG boundary (parallel to the loading direction) in the QT sample (Figure 7(d2)). The higher proportion of HAGBs in the MTP sample also provided more effective obstacles to cleavage crack propagation, so higher cryogenic impact toughness was obtained.

## 6. Conclusions

In this study, we evaluated tensile properties and impact toughness at cryogenic temperatures for medium carbon 25Cr2Ni3MoV steel by comparing (i) conventional quenching and tempering (QT) and (ii) multi-step tempering (MTP).

Compared to the QT sample, the MTP sample possesses refined martensite lath and cementite through the reduction in tempering time and temperature, and corresponding excellent cryogenic results were obtained for yield strength (1300 MPa), total elongation (25%), and impact toughness (>25 J) at liquid nitrogen temperature.

The high interface density and refinement of cementite along the PAG boundary were obtained through the use of reversion martensite transformation and austenite decomposition during the MTP process, improving the strength and coordinating the deformation compatibility by means of dispersive strain concentration bands.

Moreover, coarse cementite along HAGBs will promote local strain concentration and microvoid nucleation and weak PAG boundaries’ ability to consume crack propagation energy, which is a fatal threat to ductile metal material.

## Figures and Tables

**Table 1:** Element content of 25Cr2Ni3MoV steel.

Element (wt.%)	C	Ni	Cr	Mo	Mn	Si	V	Fe
Content	0.25	2.8–3.1	1.4–1.6	0.51	0.23	0.21	0.13	Balanced

**Table 2:** Parameters for the crystal plasticity of tempering martensite.

Crystal Parameters		QT		MTP	
		{112}<111>	{110}<111>	{112}<111>	{110}<111>
G/GPa	C11	231	231	231	231
	C12	134	134	134	134
	C44	116	116	116	116
h_0_/MPa		1100	1100	1300	1300
τ_s_/MPa		300	300	320	320
τ_0_/MPa		250	250	270	270
q		1	1	1	1
n		20	20	20	20
${\dot{\gamma }}_{0}/s^{-1}$		0.001	0.001	0.001	0.001

Elastic modulus (C11, C12, and C44), initial hardening modulus (h0), saturation stress (τ_s_), yield stress (τ_0_), hardening constant (q), strain rate sensitivity coefficient (n), and the referring shear strain rate (${\dot{\gamma }}_{0}$).

**Table 3:** The mechanical properties of the two types of steel.

Sample	YS (MPa)	TS (MPa)	ETF (%)	A_K_ (J)						H (HRC)
	−196 °C	−196 °C	−196 °C	−150 °C	−160 °C	−170 °C	−180 °C	−190 °C	−196 °C	
QT	1226.6 ± 10.1	1283.2 ± 11.4	18.1 ± 1.1	34.1 ± 4.2	26.6 ± 3.3	19.4 ± 2.5	15.6 ± 3.1	9.1 ± 1.7	6.3 ± 1.5	304 ± 1.7
MTP	1332.1 ± 15.3	1367.7 ± 16.1	25.4 ± 0.7	69.3 ± 2.3	62.9 ± 2.7	50.8 ± 2.1	37.9 ± 1.6	30.6 ± 1.7	24.5 ± 1.1	325 ± 2.5

TS, tensile strength; YS, yield strength; ETF, elongation to fracture; H: hardness; A_K_: impact toughness.

**Table 4:** Strength contribution for the two types of steel at room temperature.

Sample	σ_INT_	*d*	σ_GB_	$\overset{‾}{d}$	f	σ_PRE_	ρ	σ_DIS_	σ_Totall(cal)_	σ_total(exp)_
	MPa	μm	MPa	nm	%	MPa	×10^−14^ m^−2^	MPa	MPa	MPa
QT	160	4	142	120	7	120	2.38	307	729	746
MTP	160	1.5	168	80	4	138	3.51	380	826	820

## Data Availability

The data presented in this study are available upon request from the corresponding author.
